# Long-term renal outcomes in patients with traumatic brain injury: A nationwide population-based cohort study

**DOI:** 10.1371/journal.pone.0171999

**Published:** 2017-02-14

**Authors:** Chia-Lin Wu, Chew-Teng Kor, Ping-Fang Chiu, Chun-Chieh Tsai, Ie-Bin Lian, Tao-Hsiang Yang, Der-Cherng Tarng, Chia-Chu Chang

**Affiliations:** 1 Division of Nephrology, Department of Internal Medicine, Changhua Christian Hospital, Changhua, Taiwan; 2 School of Medicine, Chung-Shan Medical University, Taichung, Taiwan; 3 Institute of Clinical Medicine, National Yang-Ming University, Taipei, Taiwan; 4 Internal Medicine Research Center, Changhua Christian Hospital, Changhua, Taiwan; 5 Environmental and Precision Medicine Laboratory, Changhua Christian Hospital, Changhua, Taiwan; 6 Graduate Institute of Statistics and Information Science, National Changhua University of Education, Changhua, Taiwan; 7 Division of Nephrology, Department of Medicine, Taipei Veterans General Hospital, Taipei, Taiwan; 8 Institute of Physiology, National Yang-Ming University, Taipei, Taiwan; National Center For Scientific Research Demokritos, GREECE

## Abstract

**Background:**

Traumatic brain injury (TBI) is an important cause of death and disability worldwide. The relationship between TBI and kidney diseases is largely unknown.

**Methods:**

We aimed to determine whether TBI is associated with long-term adverse renal outcomes. We performed a nationwide, population-based, propensity score-matched cohort study of 32,152 TBI patients and 128,608 propensity score-matched controls. Data were collected by the National Health Insurance Research Database of Taiwan from 2000 to 2012. Our clinical outcomes were chronic kidney disease (CKD), end-stage renal disease (ESRD) and the composite endpoint of ESRD or all-cause mortality.

**Results:**

The incidence rate of CKD was higher in the TBI than in the control cohort (8.99 *vs*. 7.4 per 1000 person-years). The TBI patients also showed higher risks of CKD (adjusted hazard ratio [aHR] 1.14, 95% confidence interval [CI] 1.08–1.20; *P* < 0.001) and composite endpoints (aHR 1.08, 95% CI 1.01–1.15; *P* = 0.022) than the control groups, but the ESRD was not significantly different between the groups. In subgroup analyses, the risks of incident CKD and composite endpoints were significantly raised in TBI patients aged < 65 years and/or without comorbidities. However, the risks of both CKD and composite outcome were little affected by the severity of TBI.

**Conclusions:**

TBI has a modest but significant effect on incident CKD and composite endpoint, but not on ESRD alone. TBI patients under 65 are at greater risk of CKD and composite outcome than their older counterparts.

## Introduction

Traumatic brain injury (TBI) is a common disorder and an important cause of death and disability, exerting substantial impact on public health. Prior to the introduction of the motorcycle helmet law in 1997, most TBIs were caused by motorcycle-related injuries [1]. TBIs may be followed by sequelae, such as headaches, cognitive dysfunction and behavioral and mental disorders, raising healthcare costs and unemployment [2].

Chronic kidney disease (CKD) is another important cause of death and disability. CKD gradually and inevitably progresses toward end-stage renal disease (ESRD) or mortality. Significant risk factors for CKD include old age, cigarette smoking, elevated blood pressure, diabetes, dyslipidemia, proteinuria and anemia [3,4]. However, despite improved care for hypertension, diabetes and hyperlipidemia, CKD prevalence has increased throughout the past two decades [5]. It is critical to identify the potential risk factors and improve the current health care system in Taiwan, where the prevalence and incidence rates of end-stage renal disease (ESRD) are the highest in the world [6].

Severe TBI patients are prone to non-neurological organ dysfunction, which worsens their outcomes [7]. A few studies have associated TBI with the development of acute kidney injury (AKI) [8–10]. Brain–kidney crosstalk, by which acute brain injury affects the kidney through increased visceral sympathetic nervous system activity and plasma catecholamines, systolic hypertension, sodium wasting or hypovolemia, has also been proposed [11]. However, the impact of TBI-associated AKI on long-term renal outcomes has not been investigated.

Whether TBI increases the risk of adverse long-term renal outcomes (CKD, ESRD and death) is largely unknown. In particular, the impact of TBI on CKD risk has not been reported to our knowledge. Therefore, using a propensity-matching method, we performed a retrospective study relating the effects of TBI to the development of CKD, ESRD and death in a large-scale nationwide cohort.

## Materials and methods

### Data source

Patients’ data were retrieved from Taiwan’s National Health Insurance Research Database (NHIRD), which contains health-care claims data from the National Health Insurance (NHI) program from 1996 to 2012. The claims in NHIRD include the demographics, clinical visit records, ambulatory care, hospital admissions, disease status, drug prescriptions, medical procedures and dental services of approximately 23 million people (>99% of Taiwan’s population) [12]. Diseases were identified by the International Classification of Diseases, Ninth Revision, Clinical Modification (ICD-9-CM) codes. Because the analyzed information was de-identified and encrypted, this study was exempted from full review and approved by the Institutional Review Board of the Changhua Christian Hospital (approval number 150925).

### Study population

We identified newly diagnosed TBI patients (ICD-9-CM codes 800, 801, 803, 804, 850–854) from January 1 2000 to December 31 2012. The index date was defined as the first date of TBI diagnosis. Patients who had developed AKI (ICD-9-CM codes 584.3, 634.3, 635.3, 636.3, 637.3, 638.3, 639.3, 669.3, 958.5), CKD and ESRD (ICD-9-CM codes 580–589) before the index date, those aged <18 years or >100 years, those who survived for < 30 days after TBI diagnosis, those who were followed for < 30 days, and those with incomplete demographic information, were excluded from the study. Each identified TBI patient was matched with four control subjects by propensity scoring (S1 Fig).

### Outcomes and relevant variables

Our first primary outcome was CKD. To this end, we followed TBI and non-TBI cohorts from the index date to the first occurrence date of CKD (ICD-9-CM code 580–589), the date of withdrawal from the insurance system, or the end of 2012. The CKD risk is likely to be affected by demographics, major comorbidities diagnosed within 1 year of the index date, and specific drugs such as angiotensin-converting-enzyme inhibitors (ACEIs), angiotensin II receptor blockers (ARBs), non-steroidal anti-inflammatory drugs (NSAIDs), and anti-gout agents. The comorbidities included hypertension (ICD-9-CM codes 401–405), diabetes mellitus (ICD-9-CM code 250), hyperlipidemia (ICD-9-CM code 272), coronary artery disease (CAD) (ICD-9-CM codes 410–414), cardiac arrhythmia (ICD-9-CM codes 426–427, V45.0, V53.3), stroke (ICD-9-CM codes 430–438), peripheral artery occlusive disease (PAOD) (ICD-9-CM codes 443–444), anemia (ICD-9-CM codes 280–285), and gout (ICD-9-CM code 274). The baseline comorbidities were weighted by the Charlson’s comorbidity index (CCI) score [13]. We also evaluated the risks of ESRD and the composite endpoint (ESRD or all-cause mortality) between the two cohorts. ESRD was assumed in patients receiving dialysis therapy for at least 90 days [14].

### Statistical analysis

Continuous demographic and clinical characteristics were expressed as means ± standard deviation (SD), whereas discrete variables were expressed as counts and percentages. Categorical and continuous variables were compared in *χ*^2^ tests and *t* tests, respectively. To balance the measured covariate distributions in the two study cohorts, we calculated a propensity score for the TBI likelihood of each patient, using the baseline covariates in a non-parsimonious multivariate logistic regression model. Based on the propensity score, each patient in the TBI cohort was matched to four non-TBI controls. The relative risks (crude hazard ratios [cHRs] and 95% confidence intervals [CIs]) of developing CKD in the two study cohorts were determined by Cox’s proportional hazard models. Confounders (age, sex, clinical visit frequency, income and all comorbidities and medications) were adjusted by multivariate Cox’s analysis, yielding the adjusted hazard ratios (aHRs). Because ACEIs, ARBs, NSAIDs and anti-gout agents may be substantially associated with CKD and ESRD, these medications were considered as time-dependent covariates in a survival analysis. Additionally, because CKD and ESRD risks might compete with the risk of death, we performed a competing-risks regression (Fine–Gray model) based on the Cox’s proportional hazard model. We also compared the aHRs of CKD and composite endpoints among the patient subgroups. The interactive effects of TBI and other clinical characteristics on the risks of CKD and composite endpoints were evaluated by multiplicative analyses using likelihood ratio tests. We used multi-state models which permits modeling of the hazard of transitioning from one state to the possible subsequent states using the Cox’s proportional hazards with competing risks (S2 Fig). We also used a Cox’s proportional hazards model with age and severity of TBI as time-dependent covariates to account for the effects of aging and severity of TBI during follow-up on the risks of CKD. The cumulative CKD incidences in both study cohorts were examined by the Kaplan–Meier method and compared in log-rank tests. All statistical analyses were performed using SAS 9.4 software (SAS Institute Inc., Cary, NC). A two-tailed *P* value < 0.05 was considered statistically significant.

## Results

### Characteristics of patients

This study enrolled 160,760 participants, including 32,152 patients diagnosed with TBI between January 1 2000 and December 31 2012, and 128,608 propensity score-matched controls not diagnosed with TBI (S1 Fig). The mean follow-up times of these cohorts were 6.80 ± 3.79 and 6.77 ± 3.78 years, respectively. The average age, sex distribution, and proportions of patients with hypertension, hyperlipidemia, CAD, cardiac arrhythmia, stroke, PAOD, anemia and drug usage were similar in both cohorts (Table 1). The TBI patients generally had lower monthly income, more frequent clinic visits and slightly higher CCI scores and prevalence of diabetes mellitus than the control cohort, but a slightly lower proportion of gout at baseline.

**Table 1:** Baseline characteristics and clinical outcomes of study patients after propensity score matching.

Variables^a^	TBI (*n* = 32,152)	Non-TBI (*n* = 128,608)	*P* value^b^
Age (years)	45.9 ± 19.1	45.8 ± 19.0	0.430
Male	17636 (54.9%)	70544 (54.9%)	1.000
Monthly income (NTD)	14500.6 ± 11692.7	17199.2 ± 15646.4	<0.001
Number of outpatient visits in the past year	16.1 ± 16.9	13.4 ± 14.4	<0.001
Comorbidities			
Hypertension	5802 (18.1%)	23309 (18.1%)	0.744
Diabetes mellitus	2703 (8.4%)	9912 (7.7%)	<0.001
Hyperlipidemia	2164 (6.7%)	8993 (7.0%)	0.098
CAD	1605 (5.0%)	6701 (5.2%)	0.113
Cardiac arrhythmia	787 (2.5%)	3045 (2.4%)	0.400
Stroke	1381 (4.3%)	5500 (4.3%)	0.883
PAOD	140 (0.44%)	486 (0.4%)	0.138
Anemia	550 (1.7%)	2020 (1.6%)	0.074
Gout	1120 (3.5%)	4811 (3.7%)	0.029
CCI score	2.22±2.33	1.75±2.12	<0.001
Medications			
ACEIs or ARBs	2316 (7.2%)	9606 (7.5%)	0.104
NSAIDs	272 (0.9%)	1090 (0.9%)	0.978
Anti-gout agents	840 (2.6%)	3466 (2.7%)	0.413
Outcomes in the follow-up period			
Incident CKD	1966 (6.1%)	6448 (5.0%)	<0.001
ESRD	104 (0.32%)	365 (0.28%)	0.238
Composite endpoint^c^	1312 (4.1%)	4094 (3.2%)	<0.001

Abbreviations: ACEI, Angiotensin-converting-enzyme inhibitor; ARB, Angiotensin II receptor blocker; CAD, coronary artery disease; CCI, Charlson’s comorbidity index; CKD, chronic kidney disease; ESRD, end-stage renal disease; NSAIDs, Non-steroidal anti-inflammatory drugs; NTD, new Taiwan dollars; PAOD, peripheral artery occlusive disease; SD, standard deviation; TBI, traumatic brain injury.

^a^Variables are expressed as mean ± SD or *n* (%).

^b^2-sided *t* test or χ^2^ test between TBI and non-TBI cohorts.

^c^Composite endpoint of ESRD or an all-cause death prior to dialysis.

### Long-term risk of incident CKD

During follow-up, the proportion of TBI patients with incident CKD was 6.1% (*vs*. 5.0% in the control group, *P* < 0.001; Table 1). The incidence rate of CKD (8.99 *vs*. 7.4 per 1000 person-years; Table 2) was significantly higher in the TBI cohort than in the control cohort. The TBI patients were also at higher risk of incident CKD than the control patients (cHR, 1.21; 95% CI, 1.15–1.27; *P* < 0.001), even after adjusting for confounders (aHR, 1.14; 95% CI, 1.08–1.20; *P* < 0.001). In the subgroup analyses, the CKD risks were similarly higher in TBI patients of both sexes and outpatient visit frequency groups than in their corresponding control subgroups (aHRs, 1.12 in males and 1.09 in females, both *P* < 0.05; aHRs, 1.15 in visit frequency ≤ 12 times per year and 1.11 in visit frequency > 12 times per year, both *P* < 0.05). In contrast, among the TBI cohort, the CKD risk was raised only in patients under 65, those without comorbidities, and those with monthly incomes below 25,000 new Taiwan dollars (NTD), compared with the control cohort (all *P* < 0.05).

**Table 2:** Incidences and hazard ratios of CKD in TBI patients and non-TBI cohort, compared by demographic characteristics and comorbidities.

Variables	Subjects without TBI			Subjects with TBI			TBI cohort *vs*. Non-TBI cohort			
	Events	Person-years	Incidence^a^	Events	Person-years	Incidence^a^	cHR (95% CI)	*P* value	aHR^b^ (95% CI)	*P* value
Overall for CKD	6448	871163	7.4 (7.22–7.58)	1966	218708	8.99 (8.59–9.39)	1.21 (1.15–1.27)	<0.001	1.14 (1.08–1.20)	<0.001
Sex										
Female	2586	398999	6.48 (6.23–6.73)	776	101268	7.66 (7.12–8.2)	1.18 (1.09–1.28)	<0.001	1.09 (1.00–1.18)	0.048
Male	3862	472163.98	8.18 (7.92–8.44)	1190	117439.78	10.13 (9.56–10.71)	1.23 (1.15–1.31)	<0.001	1.12 (1.05–1.20)	0.001
Stratify age, years										
<50	1318	574236	2.3 (2.17–2.42)	503	142850	3.52 (3.21–3.83)	1.53 (1.38–1.70)	<0.001	1.23 (1.11–1.37)	<0.001
50–64	1681	167057	10.06 (9.58–10.54)	535	43157	12.4 (11.35–13.45)	1.23 (1.11–1.35)	<0.001	1.12 (1.02–1.24)	0.024
≥65	3449	129870.71	26.56 (25.67–27.44)	928	32701	28.38 (26.55–30.2)	1.06 (0.99–1.14)	0.102	1.01 (0.93–1.08)	0.885
Baseline Comorbidity										
0	2195	666663	3.29 (3.15–3.43)	742	165796	4.48 (4.15–4.8)	1.36 (1.25–1.47)	<0.001	1.21 (1.11–1.32)	<0.001
1–2	2904	164589	17.64 (17–18.29)	874	43657	20.02 (18.69–21.35)	1.13 (1.05–1.22)	0.002	1.05 (0.97–1.13)	0.255
≥3	1349	39910.98	33.8 (32–35.6)	350	9255	37.82 (33.85–41.78)	1.11 (0.99–1.25)	0.072	1.04 (0.92–1.17)	0.541
Outpatient visit frequency^c^ (times per year)										
≤12	1183	445371.98	2.66 (2.50–2.81)	267	87698.11	3.04 (2.68–3.41)	1.14 (1.00–1.30)	0.052	1.15 (1.01–1.32)	0.040
>12	5265	425791.1	12.37 (12.03–12.7)	1699	131010	12.97 (12.35–13.59)	1.05 (0.99–1.11)	0.089	1.11 (1.05–1.17)	<0.001
Monthly income (NTD)										
<15840	3796	403923	9.4 (9.1–9.7)	1120	114890	9.75 (9.18–10.32)	1.04 (0.97–1.11)	0.305	1.08 (1.01–1.16)	0.022
15840–25000	1986	278773	7.12 (6.81–7.44)	709	76497	9.27 (8.59–9.95)	1.3 (1.19–1.41)	<0.001	1.16 (1.07–1.27)	<0.001
≥25000	666	188467	3.53 (3.27–3.8)	137	27322	5.01 (4.17–5.85)	1.42 (1.18–1.7)	<0.001	1.18 (0.98–1.42)	0.085

Abbreviations: ACEI, Angiotensin-converting-enzyme inhibitor; aHR, adjusted hazard ratio; ARB, Angiotensin II receptor blocker; CAD, coronary artery disease; cHR, crude hazard ratio; CI, confidence interval; CKD, chronic kidney disease; cHR, crude hazard ratio; NSAIDs, Non-steroidal anti-inflammatory drugs; NTD, new Taiwan dollars; PAOD, peripheral artery occlusive disease; TBI, traumatic brain injury.

^a^Incidence rate per 1000 person-years.

^b^Results of multivariate analysis including age, gender, outpatient visit frequency, monthly income, comorbidities (hypertension, diabetes mellitus, hyperlipidemia, CAD, PAOD, arrhythmia, stroke, anemia and gout) and medications (ACEIs/ARBs, anti-gout agents and NSAIDs). Time-dependent covariates were the comorbidities and medications. Competing risks were CKD and death.

^c^After the onset of TBI or index date.

**Fig 1:**
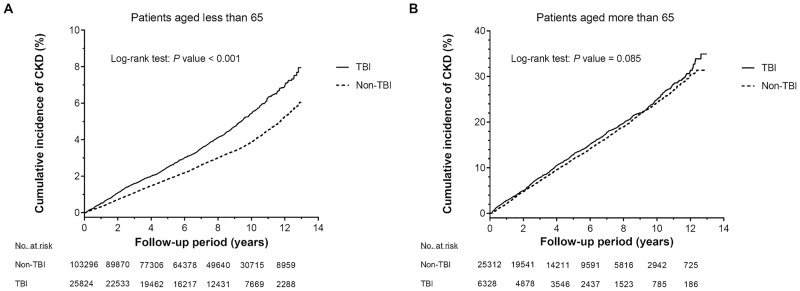
Cumulative incidence of chronic kidney disease in patients <65 (panel A) and patients aged ≥65 (panel B). Incidences of their non-TBI counterparts are shown for comparison. In A, the cumulative incidence was significantly higher in the TBI cohort than in the control cohort (*P*<0.001). In B, the cumulative incidences did not significantly differ between the TBI and non-TBI cohorts. TBI, traumatic brain injury.

**Fig 2:**
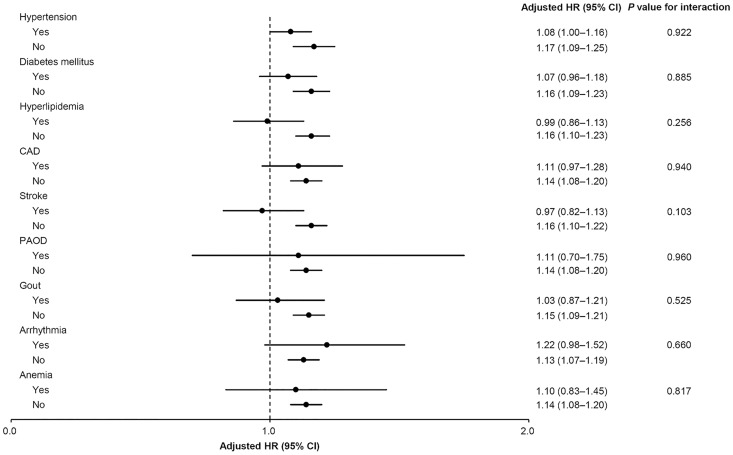
Subgroup analysis of traumatic brain injury effects on risk of chronic kidney disease.

Table 3 summarizes the interactive effects of age and TBI severity on incident CKD. Compared with the matched controls, the CKD risks were significantly increased in patients with both severe TBI (aHR, 1.17; 95% CI, 1.09–1.25; *P* < 0.001) and mild TBI (aHR, 1.12; 95% CI, 1.04–1.21; *P* = 0.003). The same effect was observed in patients under 65. According to the Kaplan–Meier survival analysis, the cumulative incidence of CKD was significantly higher in TBI patients under 65 than in the non-TBI cohort (log-rank test, *P* < 0.001) (Fig 1A). However, the association between TBI and CKD risk was absent in patients older than 65 (Table 3 and Fig 1B). The interaction between TBI and age was significant (*P* for interaction < 0.001; Table 3). Moreover, we conducted a time-varying Cox’s regression hazards model to evaluate the effect of TBI on CKD after treating age and severity of TBI as time-dependent variables (S1 Table). The analysis showed that the association between TBI and CKD was in agreement with the results in Table 3. Subgroup analyses also revealed that the aHRs of CKD increased only in patients without comorbidities (hypertension, diabetes mellitus, hyperlipidemia, CAD, stroke, PAOD, gout, arrhythmia and anemia; all *P* < 0.05), although none of these comorbidities significantly interacted with TBI (all interactions *P* > 0.05; Fig 2).

**Table 3:** Incidence rates and hazard ratios of CKD with regard to severity of TBI and patient’s age.

	Events	Person-years	Incidence^a^ (95% CI)	All patients		Age < 65		Age ≥ 65		*P* value for interaction^c^
				aHR^b^ (95% CI)	*P* value	aHR^b^ (95% CI)	*P* value	aHR^b^ (95% CI)	*P* value	
Non-TBI	6448	871163.08	7.40 (7.22–7.58)	1.00 (reference)	–	1.00 (reference)	–	1.00 (reference)	–	0.001
Mild	812	98810.58	8.22 (7.65–8.78)	1.12 (1.04–1.21)	0.003	1.14 (1.03–1.26)	0.013	0.99 (0.88–1.10)	0.793	
Severe	1154	119897.49	9.62 (9.07–10.17)	1.17 (1.09–1.25)	<0.001	1.19 (1.08–1.30)	<0.001	1.04 (0.95–1.14)	0.426	

Abbreviations: ACEI, Angiotensin-converting-enzyme inhibitor; aHR, adjusted hazard ratio; ARB, Angiotensin II receptor blocker; CAD, coronary artery disease; CI, confidence interval; CKD, chronic kidney disease; NSAIDs, Non-steroidal anti-inflammatory drugs; PAOD, peripheral artery occlusive disease; TBI, traumatic brain injury.

^a^Incidence rate, per 1000 person-years.

^b^Results of multivariate analysis including age, gender, outpatient visit frequency, monthly income, comorbidities (hypertension, diabetes mellitus, hyperlipidemia, CAD, PAOD, arrhythmia, stroke, anemia and gout) and medications (ACEIs/ARBs, anti-gout agents and NSAIDs). Time-dependent covariates were the comorbidities and medications. Competing risks were CKD and death.

^c^Likelihood ratio test for the interactive effects of TBI and age.

### Long-term risks of ESRD and composite endpoint of ESRD and all-cause mortality

The risk of composite endpoint was higher in the TBI patients than in the control cohort (aHR, 1.08; 95% CI, 1.01–1.15; *P* < 0.022; S2 Table). However, TBI was not significantly associated with ESRD. Similar to the CKD risks, the risks of composite endpoint were significantly higher in patients with both severe TBI (aHR, 1.20; 95% CI, 1.11–1.30; *P* < 0.001) and mild TBI (aHR, 1.16; 95% CI, 1.06–1.27; *P* = 0.002; S3 Table). Based on the results of multi-state model (S4 Table), TBI increased the hazard of CKD (aHR 1.15, 95% CI 1.09–1.21; *P* <0.001), all-cause mortality (aHR 1.09, 95% CI 1.01–1.18; *P* = 0.024) and progression from CKD to death (aHR 1.12, 95% CI 1.01–1.24; *P* = 0.043). Among patients younger than 65, the aHRs of the composite endpoints were similar in the mild and severe TBI subgroups (both *P* < 0.05). In addition, according to the adjusted survival curves, TBI and composite outcome were associated only in the younger subgroup (S4 Fig). In the subgroup analyses of S3 Fig, the aHRs of composite outcome were increased (relative to the corresponding control subgroups) only in patients without comorbidities, similarly to the subgroup analyses of CKD (all *P* < 0.05).

## Discussion

This study of 160,760 patients demonstrates modest but significant associations between TBI and incident CKD and composite endpoint of ESRD and all-cause mortality. However, no significant association was found between TBI and ESRD alone. The increased HR of CKD appeared in patients younger than 65 and in patients without major comorbidities. Furthermore, TBI exerted similar effects on CKD and composite outcome. The risk increases of both CKD and composite outcome were insensitive to the TBI severity.

Evidence supporting brain–kidney crosstalk has grown over the past decade [11,15]. Acute cerebral injuries can lead to cerebral salt wasting and excess secretion of antidiuretic hormone, resulting in hyponatremia. Acute cerebral injury can also exaggerate sympathetic nervous activity and increase plasma catecholamine levels, leading to systolic hypertension and reduced renal perfusion [11]. Moreover, the injured brain is subject to posttraumatic neuroinflammation, including complement activation and release of proinflammatory chemokines and cytokines [16–18]. After a brain injury, these proinflammatory mediators can leak through the dysfunctional blood–brain barrier into the systemic circulation [19]. Moreover, kidney donation after a brain death has been associated with neurohormonal activation, proinflammatory activation and increased rates of acute allograft rejection and delayed graft function [20–22]. Furthermore, TBI patients may have risk factors of acute kidney injury (AKI), such as blood loss from trauma, exposure to contrast media and nephrotoxic sepsis antibiotics, as well as the risk factors of CKD, including the use of NSAIDs [11]. These findings imply that acute brain injury exerts short-term and possible long-term deleterious effects on the kidneys. In the current study, we identified TBI as a modest but independent risk factor for CKD and composite endpoint. Our results support the hypothesis that TBI increases the long-term risk and mortality of kidney diseases. TBI survivors might benefit from renal function monitoring for early detection, and education on CKD prevention.

TBI significantly increases the mortality risk. This risk accumulates over time, especially in moderate to severe cases of TBI [23,24]. Even mild TBI can significantly reduce the long-term survival [23]. TBI patients might die before developing ESRD. After adjusting for confounders, the ESRD risk was not increased in TBI patients, but the risk of composite outcome was significantly higher in TBI patients than in the control cohort. Because the event rate of ESRD was low in both cohorts, competing risks analyses might lack sufficient power to detect the true effect [25]. Therefore, the competing risk analyses should be supplemented by survival analyses on composite endpoints.

A few studies have reported the coexistence or later development of AKI in patients with moderate to severe TBI [8–10]. The incidence of AKI in hospitalized patients is 9.2–23%. In addition, AKI greatly increases the in-hospital mortality of TBI patients (42.1–55%, *vs*. 11–18.1% in TBI patients without AKI) [9.10]. Although AKI is an important contributor to CKD and ESRD [26], TBI patients with AKI might die before reaching these stages. Therefore, the long-term risks of CKD and ESRD could be underestimated in this population.

In the current study, the CKD risk and composite endpoints in TBI patients significantly interacted with age (Table 3 and S3 Table). Younger TBI patients (<65 years) were at greater risk of incident CKD and composite outcome than patients aged 65 or over (Table 2, Fig 1 and S4 Fig). Younger patients may have a longer exposure time to TBI, exacerbating the effect on the kidneys and exaggerating the likelihood of developing kidney diseases. In addition, younger TBI patients are less likely to have comorbidities, possibly confounding the relationship between TBI and CKD or composite endpoints [27]. Therefore, TBI patients younger than 65 are at greater risk of CKD and composite outcome. Although TBI showed differential effects across the hypertension, diabetes mellitus and hyperlipidemia subgroups, the interactions were insignificant, regardless of the CKD or composite endpoint risks (Fig 2 and S3 Fig). In other words, there was no strong evidence of differential effects of TBI among these subpopulations. Further studies are needed to confirm these findings.

The large-size, longitudinal population-based data in this study are nationally representative. Consequently, the selection bias was substantially reduced and our results are quite generalizable. However, these strengths are tempered by some limitations. First, the NHIRD excludes detailed information on smoking history, body mass index, family history of renal diseases, blood pressure, lipid profile, glucose and uric acid concentrations or proteinuria, and use of over-the-counter drugs or herbal remedies. Although we performed propensity score matching and adjusted for various confounders, these unmeasured confounders might have modified our results. Second, renal outcomes were mainly identified from the ICD-9-CM codes. Because the CKD severity and estimated glomerular filtration rate were unknown, the number of patients with CKD and ESRD could be underestimated. However, ICD-9-CM codes are recognized as reliable indicators of CKD, ESRD and comorbidities [14,28,29,30]. Third, we excluded patients who survived less than 30 days after TBI diagnosis (n = 555) because death at this early period was probably due to fatal TBI and/or severe complications during hospitalization. These conditions could also lead to adverse renal outcomes. Thus, we believe that exclusion of these patients would underestimate the risk of the primary and secondary outcomes. Finally, as most of Taiwan’s population is ethnically Chinese, our results might not be applicable to other ethnic populations.

In conclusion, this large-scale retrospective cohort study reveals that TBI is a significant and independent risk factor for CKD and composite outcomes, although the effect is modest. Our findings suggest that routine screening for CKD, long-term monitoring of renal function, and interventions for preventing CKD may benefit TBI patients, even those aged below 65 years and without comorbidities. The underlying mechanism of this association, and therapeutic interventions to improve the renal outcomes and mortality of TBI patients, need clarifying in future studies.

## Supporting information

S1 Table The time-varying Cox’s regression hazards model for the risk of CKD with regard to severity of TBI and patient’s age. (DOCX)

S2 Table Incidences and hazard ratios of ESRD and composite endpoint in TBI patients and the non-TBI cohort. (DOCX)

S3 Table Incidence rates and hazard ratios of composite endpoint with regard to TBI severity and patients’ age. (DOCX)

S4 Table Multi-state models for incident CKD, ESRD, and all-cause mortality. (DOCX)

S1 Fig Flowchart of patient selection. (DOCX)

S2 Fig A flow diagram for multi-state models in the study. (DOCX)

S3 Fig Subgroup analysis of TBI effects on the risk of composite outcome of end-stage renal disease and all-cause mortality. (DOCX)

S4 Fig Adjusted survival curves of ESRD-free survival in patients younger than <65 (panel A) and patients aged ≥65 (panel B). (DOCX)
