# Supplementary material for: Long-term renal outcomes in patients with traumatic brain injury: A nationwide population-based cohort study
Source: PLoS One. 2017 Feb 14;12(2):e0171999. doi: 10.1371/journal.pone.0171999 (PMC5308784; doi:10.1371/journal.pone.0171999)
Supplement: S1 Table — (DOCX) [file pone.0171999.s001.docx]

**S1 Table.** The time-varying Cox’s regression hazards model for the risk of CKD with regard to severity of TBI and patient’s age^a^

| Variables | All patients | |  | Age < 65 | |  | Age ≥ 65 | |  |
| --- | --- | --- | --- | --- | --- | --- | --- | --- | --- |
|  | aHR^b^ (95% CI) | *P* value |  | aHR^b^ (95% CI) | *P* value |  | aHR^b^ (95% CI) | *P* value |  |
| Non-TBI | 1.00 (reference) | − |  | 1.00 (reference) | − |  | 1.00 (reference) | − |  |
| Mild TBI | 1.17 (1.08−1.27) | <0.001 |  | 1.35 (1.20−1.52) | <0.001 |  | 1.04 (0.92−1.17) | 0.566 |  |
| Severe TBI | 1.24 (1.13−1.37) | <0.001 |  | 1.50 (1.30−1.73) | <0.001 |  | 1.15 (1.01−1.30) | 0.036 |  |
| Age (year) | 1.004 (1.003−1.005) | <0.001 |  | 1.01 (1.01−1.02) | <0.001 |  | 1.02 (1.01−1.02) | <0.001 |  |

Abbreviations: ACEI, Angiotensin-converting-enzyme inhibitor; aHR, adjusted hazard ratio; ARB, Angiotensin II receptor blocker; CAD, coronary artery disease; CI, confidence interval; CKD, chronic kidney disease; ESRD, end-stage renal disease; NSAIDs, Non-steroidal anti-inflammatory drugs; PAOD, peripheral artery occlusive disease; TBI, traumatic brain injury.

^a^Severity of TBI and age were handled as time-dependent covariates.

^b^Results of multivariate analysis including age, gender, outpatient visit frequency, monthly income, comorbidities (hypertension, diabetes mellitus, hyperlipidemia, CAD, PAOD, arrhythmia, stroke, anemia and gout) and medications (ACEIs/ARBs, anti-gout agents and NSAIDs).
