# Supplementary material for: Long-term renal outcomes in patients with traumatic brain injury: A nationwide population-based cohort study
Source: PLoS One. 2017 Feb 14;12(2):e0171999. doi: 10.1371/journal.pone.0171999 (PMC5308784; doi:10.1371/journal.pone.0171999)
Supplement: S2 Table — (DOCX) [file pone.0171999.s002.docx]

**S2 Table.** Incidences and hazard ratios of ESRD and composite endpoint^a^ in TBI patients and the non-TBI cohort

|  | ESRD | | | | |  | ESRD or Death^a^ | | | | |
| --- | --- | --- | --- | --- | --- | --- | --- | --- | --- | --- | --- |
|  | Events | Person- years | Incidence^b^ (95% CI) | aHR^c,d^ (95% CI) | *P* value |  | Events | Person- years | Incidence^b^ (95% CI) | aHR^c^ (95% CI) | *P* value |
| Non-TBI | 365 | 871163.1 | 0.42 (0.38−0.46) | 1.00 (reference) | − |  | 4095 | 871163.1 | 4.70 (4.56−4.84) | 1.00 (reference) | − |
| TBI | 104 | 218708.1 | 0.48 (0.38−0.57) | 0.93 (0.74−1.16) | 0.510 |  | 1312 | 218708.1 | 6.00 (5.67−6.32) | 1.08 (1.01−1.15) | 0.022 |

Abbreviations: ACEI, Angiotensin-converting-enzyme inhibitor; aHR, adjusted hazard ratio; ARB, Angiotensin II receptor blocker; CAD, coronary artery disease; CI, confidence interval; ESRD, end-stage renal disease; NSAIDs, Non-steroidal anti-inflammatory drugs; PAOD, peripheral artery occlusive disease; TBI, traumatic brain injury.

^a^A composite endpoint of ESRD or all-cause death prior to dialysis.

^b^Incidence rate per 1000 person-years.

^c^Results of multivariate analysis including age, gender, outpatient visit frequency, monthly income, comorbidities (hypertension, diabetes mellitus, hyperlipidemia, CAD, PAOD, arrhythmia, stroke, anemia and gout) and medications (ACEIs/ARBs, anti-gout agents and NSAIDs). Time-dependent covariates were the comorbidities and medications.

^d^Competing risks were ESRD and death.
