# Supplementary material for: Long-term renal outcomes in patients with traumatic brain injury: A nationwide population-based cohort study
Source: PLoS One. 2017 Feb 14;12(2):e0171999. doi: 10.1371/journal.pone.0171999 (PMC5308784; doi:10.1371/journal.pone.0171999)
Supplement: S3 Table — (DOCX) [file pone.0171999.s003.docx]

**S3 Table.** Incidence rates and hazard ratios of composite endpoint^a^ with regard to TBI severity and patients’ age

|  | Events | Person-years | Incidence^b^ (95% CI) | All patients | |  | Age < 65 | |  | Age ≥ 65 | |  | *P* value  for interaction^d^ |
| --- | --- | --- | --- | --- | --- | --- | --- | --- | --- | --- | --- | --- | --- |
|  |  |  |  | aHR^c^ (95% CI) | *P* value |  | aHR^c^ (95% CI) | *P* value |  | aHR^c^ (95% CI) | *P* value |  |  |
| Non-TBI | 4095 | 871163.08 | 4.7 (4.56−4.84) | 1.00 (reference) | − |  | 1.00 (reference) | − |  | 1.00 (reference) | − |  | <0.001 |
| Mild | 532 | 98810.58 | 5.38 (4.93−5.83) | 1.16 (1.06−1.27) | 0.002 |  | 1.16 (1.00−1.34) | 0.044 |  | 0.98 (0.87−1.10) | 0.718 |  |  |
| Severe | 780 | 119897.49 | 6.51 (6.06−6.96) | 1.20 (1.11−1.30) | <0.001 |  | 1.19 (1.05−1.34) | 0.008 |  | 0.94 (0.85−1.03) | 0.198 |  |  |

Abbreviations: ACEI, Angiotensin-converting-enzyme inhibitor; aHR, adjusted hazard ratio; ARB, Angiotensin II receptor blocker; CAD, coronary artery disease; CI, confidence interval; ESRD, end-stage renal disease; NSAIDs, Non-steroidal anti-inflammatory drugs; PAOD, peripheral artery occlusive disease; TBI, traumatic brain injury.

^a^A composite endpoint of ESRD or all-cause death prior to dialysis.

^b^Incidence rate per 1000 person-years.

^c^Results of multivariate analysis including age, gender, outpatient visit frequency, monthly income, comorbidities (hypertension, diabetes mellitus, hyperlipidemia, CAD, PAOD, arrhythmia, stroke, anemia and gout) and medications (ACEIs/ARBs, anti-gout agents and NSAIDs). Time-dependent covariates were the comorbidities and medications.

^d^Likelihood ratio test for the interactive effects of TBI and age.
