# Supplementary material for: Long-term renal outcomes in patients with traumatic brain injury: A nationwide population-based cohort study
Source: PLoS One. 2017 Feb 14;12(2):e0171999. doi: 10.1371/journal.pone.0171999 (PMC5308784; doi:10.1371/journal.pone.0171999)
Supplement: S4 Table — (DOCX) [file pone.0171999.s004.docx]

**S4 Table.** Multi-state models for incident CKD, ESRD, and all-cause mortality

| Variables | Scenario 1 | | | | | | | |  | Scenario 2 | | | | |
| --- | --- | --- | --- | --- | --- | --- | --- | --- | --- | --- | --- | --- | --- | --- |
|  | CKD^a^ | |  | ESRD^a^ | |  | All-cause mortality | |  | From CKD to ESRD^a^ | |  | From CKD to death | |
|  | aHR^b^  (95% CI) | *P* value |  | aHR^b^  (95% CI) | *P* value |  | aHR^b^  (95% CI) | *P* value |  | aHR^b^  (95% CI) | *P* value |  | aHR^b^  (95% CI) | *P* value |
| Non-TBI | 1.00 (reference) | — |  | 1.00 (reference) | — |  | 1.00 (reference) | — |  | 1.00 (reference) | — |  | 1.00 (reference) | — |
| TBI | 1.15 (1.09−1.21) | <0.001 |  | 0.58 (0.2−1.72) | 0.324 |  | 1.09 (1.01−1.18) | 0.024 |  | 0.88 (0.7−1.1) | 0.273 |  | 1.12 (1.01−1.24) | 0.043 |

Abbreviations: ACEI, Angiotensin-converting-enzyme inhibitor; aHR, adjusted hazard ratio; ARB, Angiotensin II receptor blocker; CAD, coronary artery disease; CI, confidence interval; CKD, chronic kidney disease; ESRD, end-stage renal disease; NSAIDs, Non-steroidal anti-inflammatory drugs; PAOD, peripheral artery occlusive disease; TBI, traumatic brain injury.

^a^Competing risk of death.

^b^Results of multivariate analysis including age, gender, outpatient visit frequency, monthly income, comorbidities (hypertension, diabetes mellitus, hyperlipidemia, CAD, PAOD, arrhythmia, stroke, anemia and gout) and medications (ACEIs/ARBs, anti-gout agents and NSAIDs). Time-dependent covariates were the comorbidities and medications.
