# Supplementary material for: Long-term renal outcomes in patients with traumatic brain injury: A nationwide population-based cohort study
Source: PLoS One. 2017 Feb 14;12(2):e0171999. doi: 10.1371/journal.pone.0171999 (PMC5308784; doi:10.1371/journal.pone.0171999)

**S1 Fig.** Flowchart of patient selection. NHIRD, National Health Insurance Research Database; TBI, traumatic brain injury.


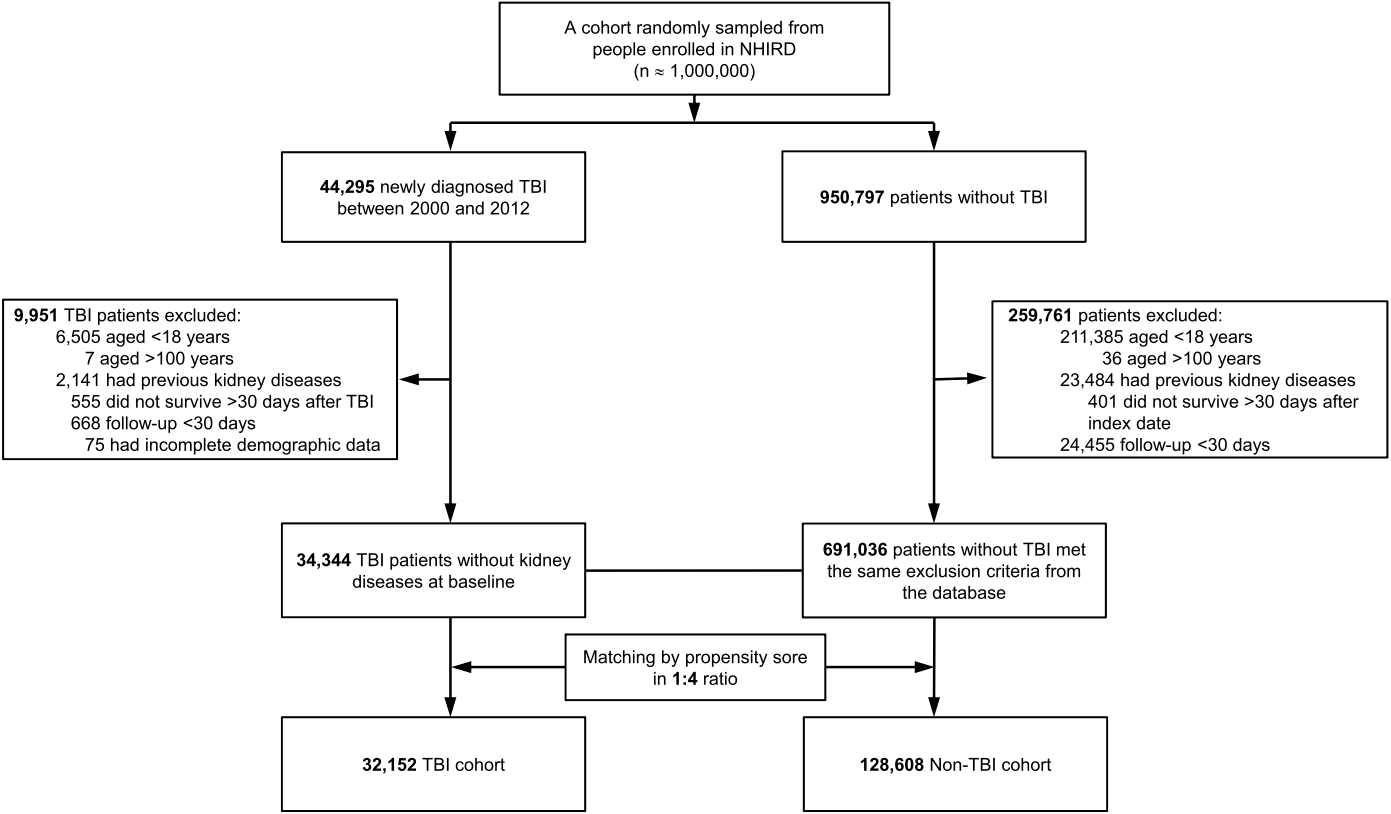

Supplement: S1 Fig — (DOCX) [file pone.0171999.s005.docx]
