# Supplementary material for: Long-term renal outcomes in patients with traumatic brain injury: A nationwide population-based cohort study
Source: PLoS One. 2017 Feb 14;12(2):e0171999. doi: 10.1371/journal.pone.0171999 (PMC5308784; doi:10.1371/journal.pone.0171999)
Supplement: S2 Fig — (DOCX) [file pone.0171999.s006.docx]

**S2 Fig**. A flow diagram for multi-state models in the study.


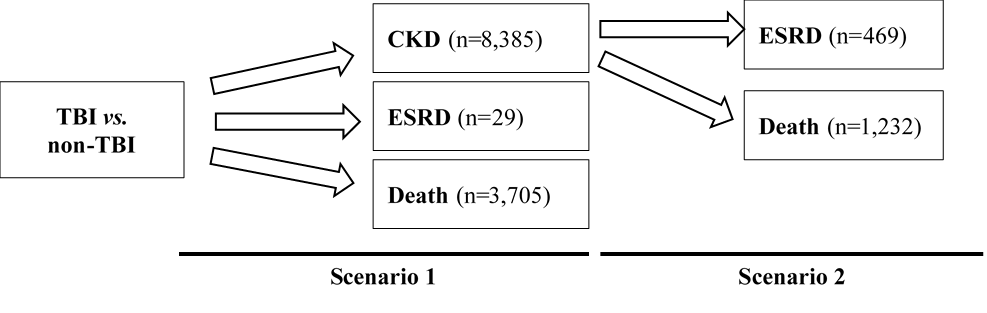


CKD, chronic kidney disease; ESRD, end-stage renal disease; TBI, traumatic brain injury.
