# Supplementary material for: Long-term renal outcomes in patients with traumatic brain injury: A nationwide population-based cohort study
Source: PLoS One. 2017 Feb 14;12(2):e0171999. doi: 10.1371/journal.pone.0171999 (PMC5308784; doi:10.1371/journal.pone.0171999)
Supplement: S4 Fig — (DOCX) [file pone.0171999.s008.docx]

**S4 Fig**. Adjusted survival curves of ESRD-free survival in patients younger than <65 (panel A) and patients aged ≥65 (panel B). Incidences of their non-TBI counterparts are shown for comparison.


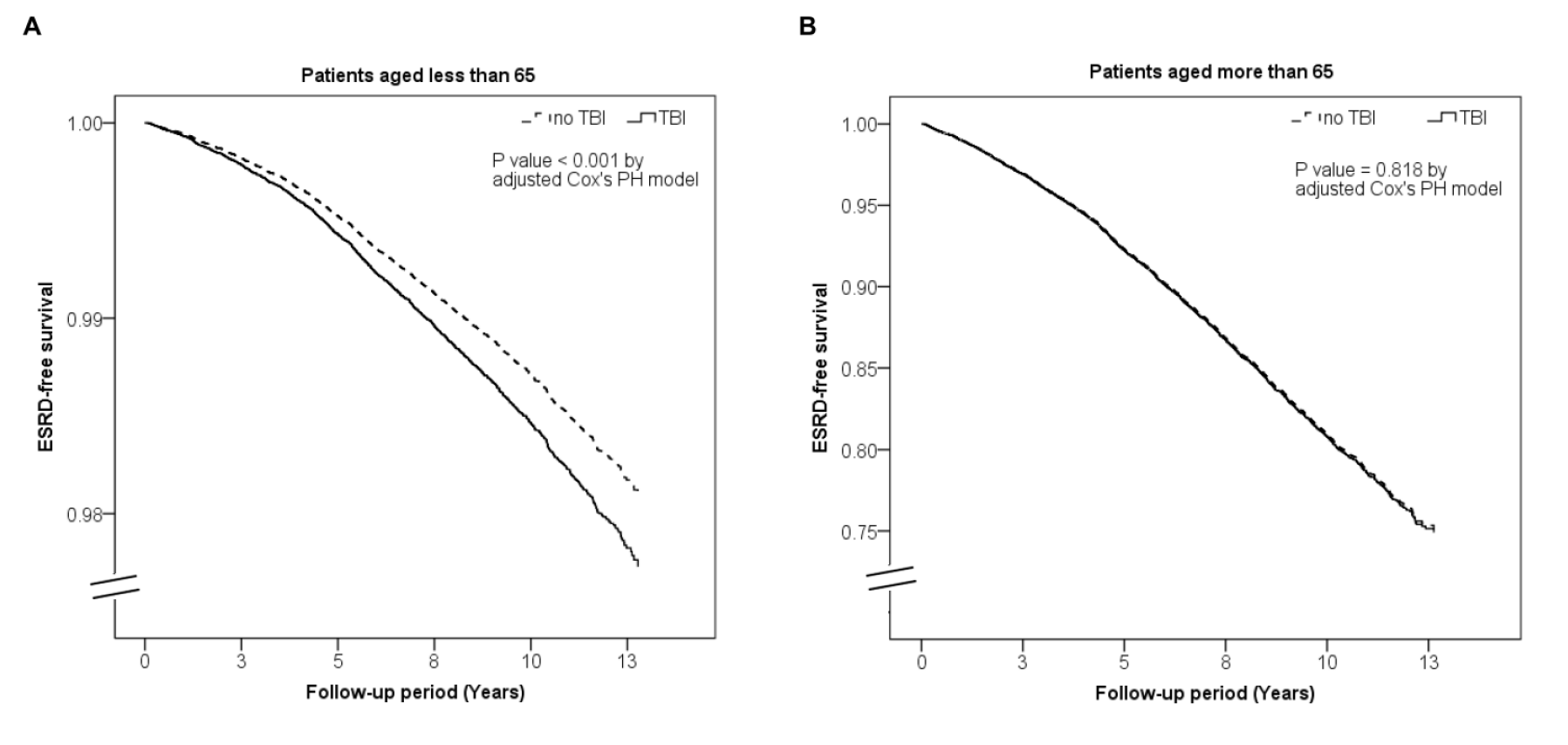


In A, the cumulative survival (adjusted for confounders by Cox’s proportional hazards model) was significantly lower in the TBI cohort than in the control cohort (*P*<0.001). In B, the adjusted cumulative survivals did not differ significantly among the two cohorts. ESRD, end-stage renal disease; TBI, traumatic brain injury.
